# High-Frequency Alternating Current Block Using Macro-Sieve Electrodes: A Pilot Study

**DOI:** 10.7759/cureus.13728

**Published:** 2021-03-06

**Authors:** Soumyajit Ray, Saad Javeed, Jawad M Khalifeh, Nikhil Chandra, Nathan Birenbaum, John M Felder, Daniel Moran, Wilson Z Ray, Matthew R MacEwan

**Affiliations:** 1 Department of Biomedical Engineering, Washington University School of Medicine, St. Louis, USA; 2 Department of Neurological Surgery, Washington University School of Medicine, St. Louis, USA; 3 Department of Plastic Surgery, Washington University School of Medicine, St. Louis, USA

**Keywords:** high frequency alternating current, macro-sieve electrode, peripheral nerve, neural prosthetics, nerve conduction block, neuromodulation, neuropathic pain

## Abstract

Background and objective

High-frequency alternating current (HFAC) can yield a rapid-acting and reversible nerve conduction block. The present study aimed to demonstrate the successful implementation of HFAC block delivery via regenerative macro-sieve electrodes (MSEs).

Methods

Dual-electrode assemblies in two configurations [dual macro-sieve electrode-1 (DMSE-I), DMSE-II] were fabricated from pairs of MSEs and implanted in the transected and subsequently repaired sciatic nerves of two male Lewis rats. After four months of postoperative nerve regeneration through the MSEs' transit zones, the efficacy of acute HFAC block was tested for both configurations. Frequencies ranging from 10 kHz to 42 kHz, and stimulus amplitudes with peak-to-peak voltages ranging from 2 V to 20 V were tested. Evoked muscle force measurement was used to quantify the nerve conduction block.

Results

HFAC stimulation delivered via DMSE assemblies obtained a complete block at frequencies of 14 to 26 kHz and stimulus amplitudes of 12 to 20 V p-p. The threshold voltage for the complete block showed an approximately linear dependence on frequency. The threshold voltage for the partial conduction block was also approximately linear. For those frequencies that displayed both partial and complete block, the partial block thresholds were consistently lower.

Conclusion

This study provides a proof of concept that regenerative MSEs can achieve complete and reversible conduction block via HFAC stimulation of regenerated nerve tissue. A chronically interfaced DMSE assembly may thereby facilitate the inactivation of targeted nerves in cases wherein pathologic neuronal hyperactivity is involved.

## Introduction

Various neurological diseases are characterized by hyperactivity of sensory or motor neurons leading to chronic neuropathic pain or dystonia. These conditions are usually treated via blockade of nerve conduction, thereby alleviating symptoms [1]. Chemical blockade using anesthetics, or pharmaceutical nerve stabilizers are usually used for treatment; however, due to adverse effects and issues with long-term dependency, alternative non-pharmacological approaches are increasingly being preferred [2,3]. Surgical nerve destruction provides a long-term alternative but is irreversible [4]. A high-frequency alternating current (HFAC) can induce a peripheral nerve conduction block that quickly reverses after the current is turned off. A number of studies have demonstrated the use of HFAC to deliver conduction blocks with minimal adverse effects and rapid reversibility [5-10].

Numerous extraneural and intraneural electrodes have been used to exploit the benefits of HFAC block in animal models. A conduction block must be achieved at a suitably low stimulation threshold to maintain the nerve’s health and integrity with chronic use. The threshold frequency depends on the blocking electrode’s design and distance to axons [11,12]. Previous studies have shown that the greater the distance between the nerve and electrode, the greater the current intensity required to achieve the nerve block [12]. Traditionally, extraneural electrodes have been used to attain conduction blocks in nerve tissue, but they lack physical proximity to target axons required for low-stimulus thresholds [13].

**Figure 1:**
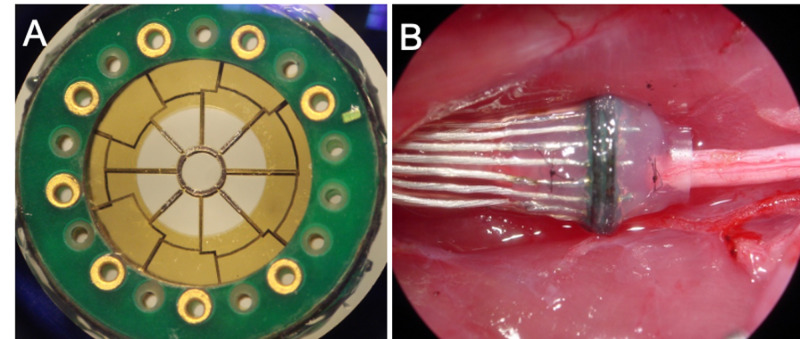
The macro-sieve electrode (MSE) A) The macro-sieve electrode (MSE) is a regenerative interface with a central porous region (diameter: 2 mm) that contains nine transit zones. After nerve transection, regenerating axons from the proximal stump pass through the transit zones and onwards to distal innervation targets. Interspersed among the transit zones are eight metalized leads that enable electrical stimulation of interfaced nerve tissue. B) Regenerated sciatic nerve successfully integrated into the MSE assembly

One alternative to extraneural electrodes is regenerative sieve electrodes. This class of electrodes relies on peripheral nerves’ innate capacity to spontaneously regenerate after transection, as observed following a nerve injury or amputation. Implantation of a regenerative electrode requires placement of the electrode between the proximal and distal stumps of a transected peripheral nerve. Regenerating axons pass through pores (i.e., “transit zones”) perforating the electrode’s surface. Interspersed among these pores is an array of electrodes that allows for highly selective and specific neural interfacing [14]. The macro-sieve electrode (MSE) (Figure 1) is a regenerative electrode variant with large transit zones and high transparency (in comparison with other variants [15,16]), which minimally impedes the passage of regenerating axons, thereby maximizing functional recovery, nerve health, and stability in the long term [17].

The aim of this study was to leverage the MSE as a unique vehicle for the delivery and achievement of HFAC nerve blockade in vivo. We hypothesized that an intimate neuron-electrode interface would require lower stimulus thresholds to achieve nerve block and increase the longevity of this modality. We employed MSEs to specifically block the fascicles under the corresponding electrode at high frequency and low amplitude. Moreover, a highly localized block would be less disruptive to surrounding axons. We believe that this pilot study will lay the groundwork for future studies that will examine the ability of MSEs to deliver the fascicle- and fiber-specific blockade necessary for targeting specific neural pathways.

## Materials and methods

DMSE construction

**Figure 2:**
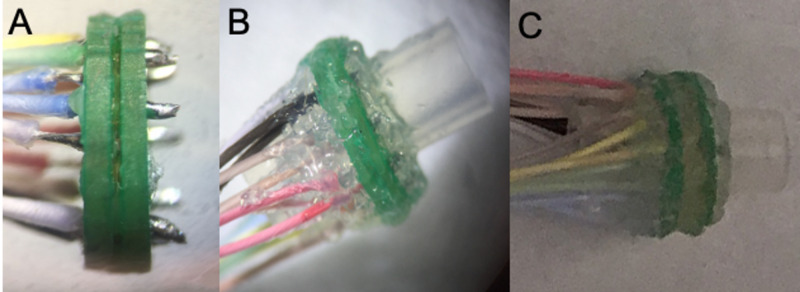
Dual macro-sieve electrode (DMSE) assembly A) DMSE-I with 0.3-mm inter-sieve distance. The Omnetics connecter microwires were soldered on either side of back-to-back MSEs. B) Completed DMSE-I assembly with silicon conduits affixed to each side. C) DMSE-II with 1-mm inter-sieve distance

The MSE fabrication was performed as described in a prior report [17]. Since the HFAC block threshold depends on the bipolar electrode distance, dual macro-sieve assemblies were fabricated in two configurations: [dual macro-sieve electrode-1 (DMSE-I), DMSE-II] (Figure 2).

DMSE-I and DMSE-II differed in the separation of their two MSEs. For DMSE-I, the MSEs were separated by a thin layer of medical-grade adhesive (Factor II, A-564 silicone adhesive) to create an inter-sieve gap of 0.3 mm. For DMSE-II, an inter-sieve gap of 1 mm was created using a segment of silicone guidance conduit (#808500, inner diameter: 0.078 in.; outer diameter 0.125 in. A-M Systems, Sequim, WA). Subsequent assembly proceeded along similar lines. Sixteen microwire leads from a male Omnetics connector (#A79044-001, Omnetics Connector Corporation, Minneapolis, MN) were soldered to the MSEs’ contacts; 2-mm sections of the silicone guidance conduit were attached to either side of the DMSE using the same medical-grade adhesive. The solder points were insulated with medical-grade epoxy (EpoTek 730, Epoxy Technologies Inc., Billerica, MA). Before surgery, the Omnetics connector terminal was wrapped in Parafilm (Parafilm PM996 Wrap) to prevent tissue deposition on its leads post-implantation.

Surgical implantation

**Figure 3:**
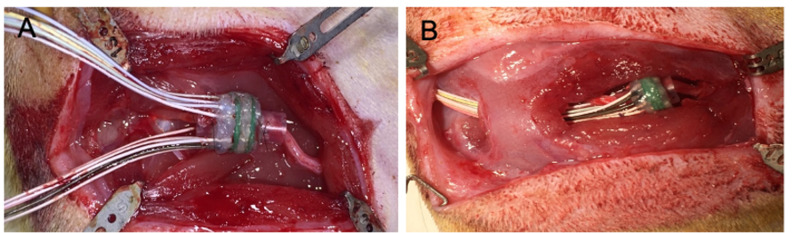
Surgical implantation of DMSE A) Following sciatic nerve transection, the severed nerve stumps were secured to the corresponding conduits using 8-0 Ethilon microsuture. B) The Omnetics connectors were tunneled under the proximal musculature routed to a dorsal subcutaneous pocket DMSE: dual macro-sieve electrode

DMSE-I and DMSE-II were implanted surgically in two adult male Lewis rats weighing 225-250 g (Figure 3). All surgical procedures were conducted using aseptic techniques and in strict accordance with regulations set forth by Washington University’s Animal Studies Committee and the Division of Comparative Medicine. The rats were anesthetized with isoflurane (5% inductance; 2% maintenance). Progressive reduction of maintenance levels over the course of surgery alleviated any detrimental effects of prolonged isoflurane exposure. The surgery proceeded in accordance with the methods described previously [18]. Briefly, the sciatic nerve was transected approximately 5 mm proximal to the trifurcation. The proximal and distal nerve stumps were positioned inside the silicone guidance conduits affixed to both sides of the DMSE. Each nerve stump was secured to its conduit using a pair of diametrically opposed microsutures (8-0 Ethilon) (Figure 3). Prior to the final suture application, the air inside the conduits was purged with saline. The DMSE’s microwire leads and attached Omnetics connector were tunneled under adjacent musculature and stored in a dorsal subcutaneous pocket. This prevented the microwires from dislodging the DMSE from the nerve and disrupting nerve regeneration; 5-0 Vicryl suture was used to close the muscle and 4-0 nylon suture was used to close the skin. Antibiotic ointment was applied topically on the area of the incision to prevent infection.

Experimental setup

Four months postoperatively, implanted rats underwent a non-survival surgery that exposed the sciatic nerve and distal musculature in order to assess the efficacy of the HFAC nerve block. A bipolar cuff electrode placed proximally to the DMSE was connected to an MS-16 stimulus isolator and RX7 base station (Tucker-Davis Technologies, Alachua, FL). This electrode delivered excitatory stimuli to the sciatic nerve. The base station was programmed to generate monophasic, rectangular current pulses at 1-Hz frequency. The pulses had an amplitude of 1 mA and a width of 0.2 ms (muscle force measurements peaked at this width).

**Figure 4:**
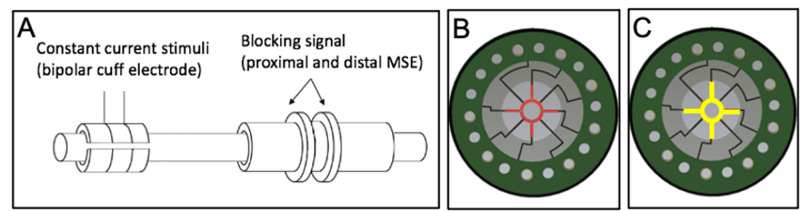
Electrode configuration in DMSE-I and DMSE-II A) Proximal stimuli were delivered by a bipolar cuff electrode. A sinusoidal blocking signal passed between the proximal and distal MSEs. B) Proximal MSE. All eight central electrode sites (in red) were used to deliver the HFAC blocking signal. C) Distal MSE. All eight central electrodes (in yellow) were used as references for the blocking signal DMSE: dual macro-sieve electrode; HFAC: high-frequency alternating current

After injecting the rat subcutaneously with 3 ml of saline, the Omnetics connector was retrieved from its dorsal subcutaneous pocket and connected to a function generator (BK Precision 4003A). This delivered the HFAC blocking signal in the form of a charge-balanced sinusoidal waveform that passed from all eight of the proximal MSE’s channels to all eight of the distal MSE’s channels. All combinations of nine frequencies and nine peak-to-peak voltages were tested for a total of 81 trials. Frequencies tested were 10 kHz, 14 kHz, 18 kHz, 22 kHz, 26 kHz, 30 kHz, 34 kHz, 38 kHz, and 42 kHz. Peak-to-peak voltages tested were 4 V, 6 V, 8 V, 10 V, 12 V, 14 V, 16 V, 18 V, and 20 V. Figure 4 depicts the described experimental setup.

The evoked muscle force was measured in the tibialis anterior (TA) muscle by the following procedure. The sciatic nerve was exposed from the sciatic notch to the popliteal fossa. Next, the TA muscle was exposed by an incision along the anterior portion of the right leg. The distal tendons of the TA were exposed, isolated, and severed distally at the site of insertion into the bone. Animals were immobilized with a C-clamp, and their femoral condyles and distal tendons were anchored to thin-film load cells (S100, Strain Measurement Devices Inc., Meriden, CT), which transduced muscle forces evoked by electrical stimulation of the sciatic nerve.

Trial structure

**Figure 5:**
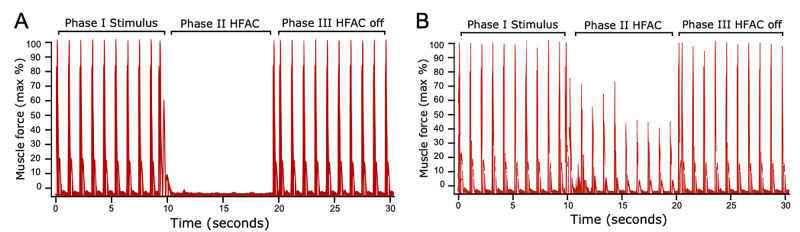
DMSE-II – HFAC block quantified by tibialis anterior muscle force measurement A) HFAC at 18 kHz and 20 V threshold demonstrating a complete block. B) HFAC at 18 kHz and 12 V threshold, a partial reduction in force measurement, demonstrating a partial block. Phase III HFAC turned off, immediate return of motor activity demonstrating reversibility of HFAC block (for frequencies and respective threshold voltages, see Figure 6) The data was filtered using a low-pass filter with a cut-off frequency of 300 Hz. The standard deviation of the baseline (without stimulus) data was determined. The peaks indicate the time points at which the signal exceeded the baseline by more than one standard deviation. Phase I stimulation started from the cuff electrode. Phase II HFAC block was switched on via DMSE DMSE: dual macro-sieve electrode; HFAC: high-frequency alternating current

Each trial had three 10-second phases. In the first phase, the stimulation of the 1-Hz current was initiated and maintained through the remaining two phases. In the second phase, HFAC stimulation was initiated. Finally, in the third phase, HFAC stimulation was terminated. Evoked muscle force measurement continued through all phases to quantify the conduction block (Figure 5).

## Results

All implanted DMSEs and silicone nerve guidance conduits survived the study duration without causing significant host tissue reactions. MSEs maintained proper placement and orientation perpendicular to the sciatic nerve. Gross observation of DMSEs confirmed the devices’ chronic stability and successful regeneration of nerve tissue through the MSEs’ transit zones. We further confirmed successful nerve regeneration through the implanted device via gross examination upon explantation at the terminal time point.

**Figure 6:**
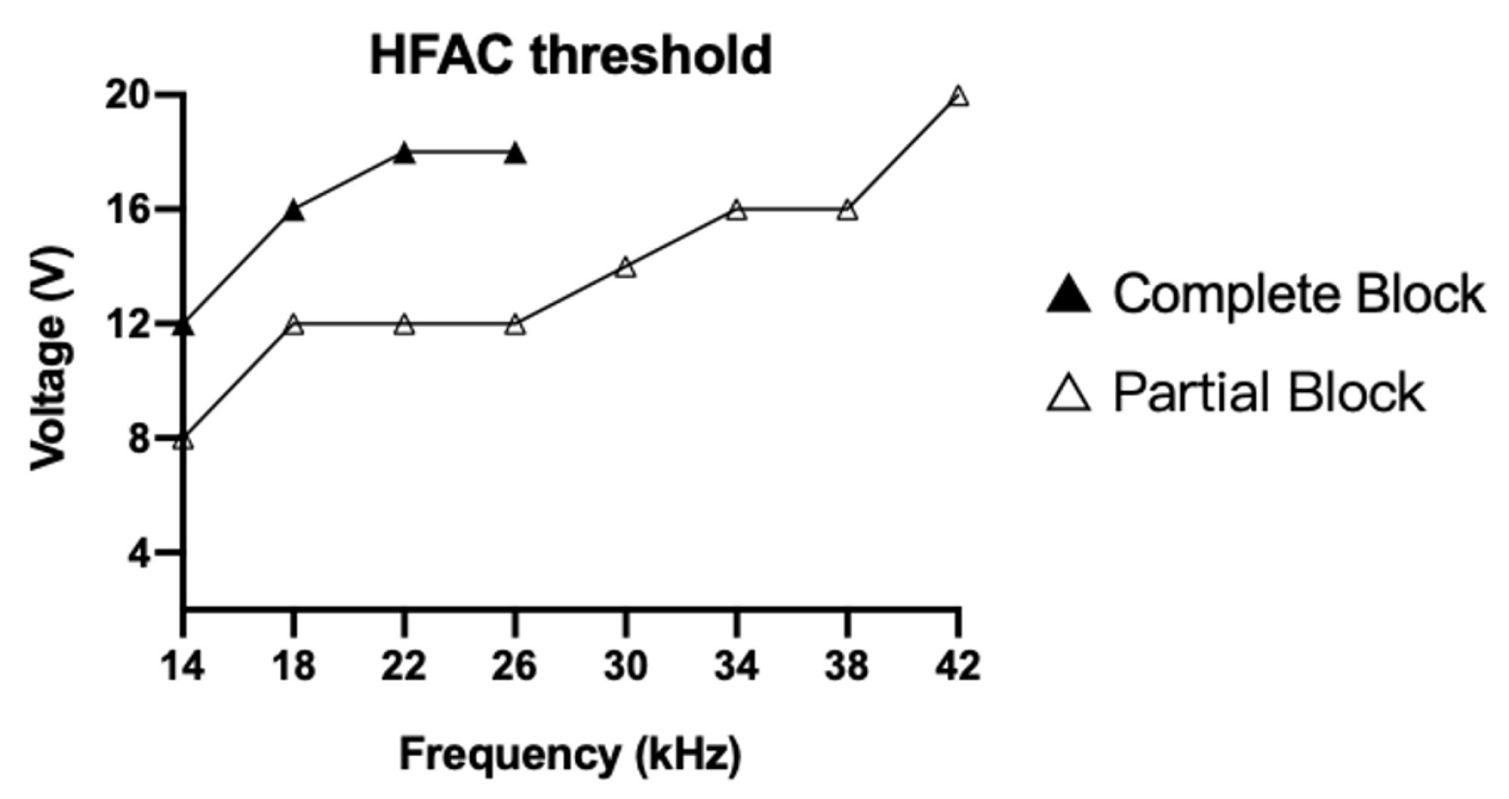
HFAC block thresholds via DMSE-II Block threshold (peak to peak voltage of HFAC blocking signal) and signal frequency. Note a linear relationship of block threshold voltages with frequencies DMSE: dual macro-sieve electrode; HFAC: high-frequency alternating current

The DMSE-I assembly (0.3-mm inter-sieve distance) failed to deliver HFAC block at any given voltage and frequency. In contrast, the DMSE-II assembly (1-mm inter-sieve distance) successfully delivered a complete and quickly reversible block for frequencies of 14-26 kHz and peak-to-peak voltages of 12-20 V (Figure 6). Additionally, frequency/voltage combinations between 14-42 kHz and peak-to-peak voltages of 8-20 V produced a partial block. The complete block threshold increased in an approximately linear fashion with the frequency. The partial block threshold also showed approximate linearity and was consistently lower than the complete block threshold.

## Discussion

This study demonstrated the successful initiation of a quickly reversible HFAC nerve block using sinusoidal waveforms passed between the proximal and distal units of a DMSE assembly. The peak-to-peak voltage threshold necessary to achieve complete block increased with frequency, and the block persisted for all amplitudes beyond the threshold. Threshold voltages for partial block also increased with frequency in an approximately linear fashion. For frequencies that exhibited both partial block and complete block, thresholds for the partial block were consistently lower than thresholds for the complete block. Evoked muscle force measurements demonstrated the DMSE’s ability to deliver a complete and reversible conduction block of regenerated nerve fibers using HFAC. Our findings reveal specific parameters of the DMSE system to achieve a successful HFAC block in a mammalian model.

An effective HFAC conduction block depends on multiple factors, including frequency, voltage, electrode geometry, the distance between bipolar blocking electrodes, and the distance from the electrodes to their target axonal population [19]. Regarding the frequency-voltage characteristic shown in Figure 6, our results are consistent with prior studies [8,9,10,20]. Bhadra and Kilgore obtained a complete conduction block at frequencies between 10-30 kHz, with a corresponding threshold voltage range of 2-10 V [20]. Similarly, experiments by Kilgore and Bhadra [8], Williamson and Andrews [10], and Bowman and McNeal [7] reported complete HFAC block over the frequency range of 2-20 kHz. Minor differences in frequency-voltage characteristics and block thresholds may be attributed to variations in stimulus parameters (current, duration), electrode type, assembly and configuration, and intrinsic properties of the peripheral nerve [21]. In the present study, frequency/voltage (p-p) combinations in the ranges of 14-26 kHz and 12-20 V completely blocked the passage of proximally generated signals. Removal of the blocking signal corresponded to a return of muscle twitches, demonstrating the immediate reversibility of the block (Figure 5). The higher threshold voltages may have multiple causes. First, newly regenerated nerve fibers crossing the MSEs’ transit zones would have smaller diameters and thinner myelin sheaths than their naïve counterparts [22]. Smaller diameter and unmyelinated fibers have previously been shown to require greater stimulation to achieve the block compared with myelinated fibers of larger diameter [23,24]. Second, significant size differences between the MSE and cuff’s metalized electrode sites may have resulted in a higher interfacial impedance, requiring a higher compliance voltage to drive current sufficient for local axon (the MSE’s individual electrode sites are only 200 nm in thickness, compared with the cuff’s 2-4 mm). The smaller-sized MSE electrode sites may therefore have required higher-threshold amplitudes to achieve the block in the surrounding axons [10,17,20].

The present study examined two DMSE configurations to determine the appropriate minimum inter-sieve distance required to achieve an effective block. DMSE-I’s inter-sieve distance was 0.3 mm; DMSE-II’s was 1 mm. While DMSE-I failed to demonstrate complete or partial nerve block, DMSE-II attained complete block for multiple combinations of frequency and voltage. Our findings are consistent with Ackermann et al.'s study, which showed that the smallest block thresholds for bipolar cuffs were associated with an inter-electrode distance of 1-2 mm [12]. They found that block thresholds increased when the inter-electrode distance rose beyond 2 mm, and also increased significantly for 0.5-mm distance. They further averred that the optimum inter-electrode spacing that would minimize the HFAC block threshold was 1.25 times the electrode-axon distance [12]. Our study’s use of MSEs, with their significantly reduced electrode-axon distance, drove our decision to test the blocking performance for an inter-sieve distance of 0.3 mm (DMSE-I). Nevertheless, the 1-mm inter-sieve distance (DMSE-II) remained the optimal spacing. Consistent with prior findings, the much-reduced inter-sieve distance of 0.3 mm failed to attain the block possibly due to spatial destruction of the electrical field generated, which was insufficient to depolarize the complete cross-sectional area of the target nerve [12].

Limitations

Our aim in this study was to establish a proof of concept that regenerative MSEs are capable of achieving rapidly reversible HFAC blocks. Our results are limited by a number of factors. Firstly, the sample size was small as it included only two rats. This was chiefly due to the complexity of device construction. Secondly, the validation of the HFAC block on sensory nerve fibers via the measurement of CNAP conduction was not performed. However, the use of evoked muscle measurement and demonstration of muscle activation immediately before and after the HFAC block confirmed that the block of motor fibers was achieved and not due to muscle fatigue. Finally, despite the intimacy of the electrode-axon interface afforded by the MSEs, relatively higher frequencies and voltages were required to achieve blocks compared with prior studies’ cuff electrodes. As mentioned previously, these unique variations are most likely attributable to the morphological parameters and average diameter of axons crossing the MSE, compared with healthy nerve tissue.

Future directions

The MSE shows significant potential as a highly selective, long-lasting interface to peripheral nerve tissue. This study demonstrated the ability of a novel dual macro-sieve assembly to achieve the HFAC block and highlighted the optimum parameters for achieving this block. Our protocol delivered the sinusoidal blocking signal using all 16 DMSE channels and therefore did not leverage the MSE’s inherent selectivity. Future studies will explore the potential of selective blockade of discrete micro-fascicles using cross-sectionally aligned electrode pairs from the proximal and distal MSEs. A successful outcome would have significant clinical implications as it would enable a selective block of nerve fibers associated with specific motor or sensory targets. Moreover, a selective block of pain fibers at higher frequencies without affecting large motor or sensory fibers may be useful in chronic pain management. The DMSE-mediated signal block and fiber selectivity may offer a unique advancement in integrated neural interfacing technology for neural prostheses and non-pharmacologic pain therapies.

## Conclusions

The DMSE assembly system is capable of delivering a complete and reversible HFAC block after nerve transection and regeneration. We established appropriate inter-sieve distance and block threshold configurations, suggesting that the efficacy of the HFAC block with DMSE depends on the exact in vivo configuration parameters. Maintenance of DMSE over the time period of the study demonstrated longevity of the stable interface for chronic neuromodulation. The intimate electrode-axon contact and the capacity for selective axonal recruitment have set the stage for exploring selective blocking of nerve fibers in the future.
